# MicroRNAs in meningiomas: Potential biomarkers and therapeutic targets

**DOI:** 10.1016/j.ncrna.2024.02.011

**Published:** 2024-02-23

**Authors:** Ozal Beylerli, Tatiana Ilyasova, Huaizhang Shi, Albert Sufianov

**Affiliations:** a Central Research Laboratory, Bashkir State Medical University, Republic of Bashkortostan, 3 Lenin Street, Ufa, 450008, Russia; b Department of Internal Diseases, Bashkir State Medical University, Republic of Bashkortostan 450008, Ufa, Russia; c Department of Neurosurgery, First Affiliated Hospital of Harbin Medical University, Harbin, Heilongjiang, China; d Educational and Scientific Institute of Neurosurgery, Рeoples’ Friendship University of Russia (RUDN University), Moscow, Russia; e Department of Neurosurgery, Sechenov First Moscow State Medical University (Sechenov University), Moscow, Russia

**Keywords:** Meningiomas, MicroRNAs, Biomarkers, Therapeutic targets, Tumorigenesis, Intracranial tumors

## Abstract

Meningiomas, characterized primarily as benign intracranial or spinal tumors, present distinctive challenges due to their variable clinical behavior, with certain cases exhibiting aggressive features linked to elevated morbidity and mortality. Despite their prevalence, the underlying molecular mechanisms governing the initiation and progression of meningiomas remain insufficiently understood. MicroRNAs (miRNAs), small endogenous non-coding RNAs orchestrating post-transcriptional gene expression, have garnered substantial attention in this context. They emerge as pivotal biomarkers and potential therapeutic targets, offering innovative avenues for managing meningiomas. Recent research delves into the intricate mechanisms by which miRNAs contribute to meningioma pathogenesis, unraveling the molecular complexities of this enigmatic tumor. Meningiomas, originating from arachnoid meningothelial cells and known for their gradual growth, constitute a significant portion of intracranial tumors. The clinical challenge lies in comprehending their progression, particularly factors associated with brain invasion and heightened recurrence rates, which remain elusive. This comprehensive review underscores the pivotal role of miRNAs, accentuating their potential to advance our comprehension of meningioma biology. Furthermore, it suggests promising directions for developing diagnostic biomarkers and therapeutic interventions, holding the promise of markedly improved patient outcomes in the face of this intricate and variable disease.

## 1 Introduction

Meningiomas, arising from the arachnoid layer of the meninges surrounding the brain and spinal cord, constitute a substantial proportion, accounting for 13–26%, of intracranial tumors [1]. These tumors have been categorized into three grades (I-III) by the World Health Organization (WHO) since 2007, with the majority being typically benign, while grade III variants exhibit a higher tendency for recurrence and increased mortality [2]. Even after surgical resection, certain meningiomas can exhibit recurrence, necessitating reoperation and raising the associated risks of morbidity and mortality [3]. While surgery is often an effective treatment for benign meningiomas, in cases where complete surgical removal is hindered by challenging locations, alternative therapeutic strategies are necessary [4]. Over the past two decades, numerous studies have aimed to gain a better understanding of the biology of meningiomas in the pursuit of novel treatments [5]. Consequently, a deeper comprehension of the biological pathways and the natural history of meningiomas may lead to the development of more effective therapies, offering the potential for a cure for these tumors [6]. The World Health Organization (WHO) classification, primarily based on morphological parameters, has yet to suggest changes in the grading of meningiomas despite significant advances in understanding the molecular mechanisms of tumorigenesis and meningioma progression [7]. However, the analysis of chromosomal regions and the study of new genes are expected to contribute to future diagnosis and prognosis [8]. Molecular markers may eventually facilitate the combination of histological and molecular criteria in the classification of meningiomas [9]. The use of global genome analysis techniques, such as microarray methods and gene expression analysis, is anticipated to expedite the development of novel therapies. Nonetheless, our knowledge of the principal pathways associated with these neoplasms remains limited. Meningiomas are characterized by well-defined cytogenetic alterations, with the most common being the deletion of the long arm (q) of chromosome 22, which is present in up to 70% of sporadic meningiomas and associated with tumor initiation. The second most prevalent alteration is the deletion of the short arm (p) of chromosome 1, found in 70% of atypical and nearly 100% of anaplastic tumors, indicating its correlation with tumor progression [10].

MicroRNAs (miRNAs), a category of small non-coding RNAs, play a crucial role in regulating gene expression at the post-transcriptional level by binding to complementary sites on target mRNAs [11]. Recent evidence challenges previous assumptions, highlighting the significant roles of miRNAs as oncogenes or tumor suppressors in various human cancers [12].

**Fig. 1:**
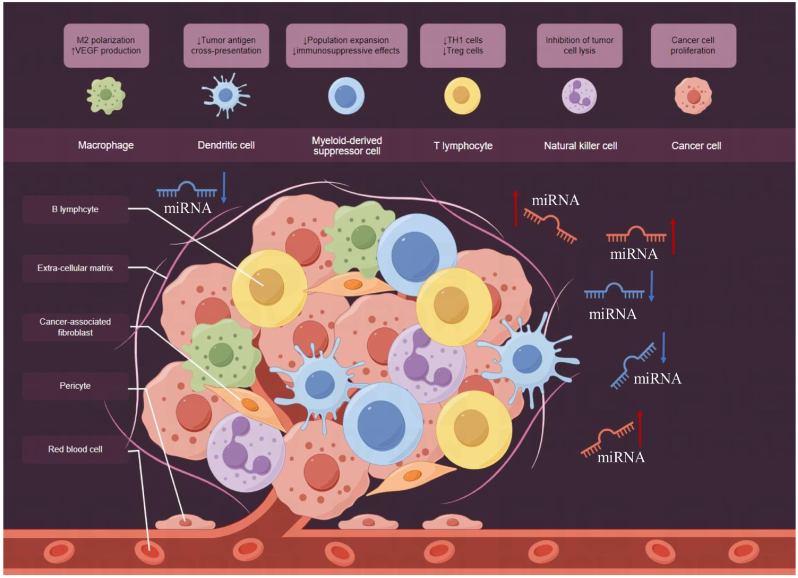
Illustrates the tumor microenvironment (TME), where tumor cell growth is supported through interactions between tumor cells and neighboring cells. The TME comprises various cell types such as tumor cells, endothelial cells, fibroblasts, and immune cells. Moreover, miRNAs play a role in regulating elements within the TME.

A previous study established a miRNA profile based on meningioma tissues, enabling risk assessment, histological classification, and predictions of postsurgical outcomes [13]. Recent discoveries emphasize the stable expression of miRNAs in human blood, raising optimism about using circulating miRNA signatures as disease-specific markers and innovative molecular biomarkers for cancer. Blood-based biomarkers offer advantages such as minimally invasiveness, high-throughput capabilities, and cost-effectiveness, making them particularly promising for large-scale applications, including in rural areas. Acting as potent post-transcriptional regulators of gene expression, miRNAs exhibit changes in expression profiles in various diseases [14]. MiRNAs remain stable in plasma, and their expression varies under different physiological and pathological conditions, positioning them as potential biomarkers [15]. Given that miRNAs are expressed at varying levels in specific tissues, and bioinformatics tools predict numerous mRNA targets for each miRNA, it is suggested that many genes undergo regulation by miRNAs. Consequently, miRNAs show promise as valuable tools for both diagnosis and prognosis. MicroRNAs play critical and diverse roles in human carcinogenesis by influencing the tumor microenvironment (TME) toward a protumor phenotype. This process, often described as a “educational” interaction between cancer cells and the surrounding TME, involves a dynamic bidirectional exchange of genetic information. Although microRNAs constitute only a portion of the extracellular cargo of vesicles, emerging scientific literature highlights their key role in shaping the microenvironment for the growth and spread of cancer cells. It is important to identify specific miRNAs and the extent to which they are involved in this process, given the differences in tumor types and miRNA concentrations used in functional studies. Understanding whether different concentrations of the same miRNA led to different target effects is critical. The idea that miRNA concentrations in the TME are systemically reflected in circulating blood levels requires further confirmation. Paracrine interactions between different TME cell populations have been studied to a limited extent, requiring the development of new 3D models for a comprehensive understanding (Fig. 1).

Additionally, there is potential for therapeutic strategies involving the manipulation of miRNAs, either through silencing or mimicking, based on their roles as oncogenes and tumor suppressor genes in brain tumors.

## 2 MiRNA biogenesis and functions

MiRNA biogenesis begins with the processing of RNA polymerase II/III transcripts, occurring concurrently or post-transcriptionally [[16], [17], [18], [19]]. A substantial number of miRNAs are derived from introns or specific protein-coding genes (intragenic), while others are intergenic, transcribed independently [[20], [21], [22], [23]]. MiRNA transcription often occurs in clusters, forming families with similar seeding regions [[24], [25], [26]]. There are two main pathways in miRNA biogenesis: canonical and non-canonical [[27], [28], [29], [30]]. In the canonical pathway, pri-miRNAs undergo processing by DGCR8 and Drosha to form pre-miRNAs, which are then transported to the cytoplasm and further processed by Dicer [[31], [32], [33]]. Mature miRNA strands are loaded into AGO proteins [34]. The non-canonical pathway involves diverse processes, including mirtron generation and the release of pre-miRNAs with m7G caps into the cytoplasm [35,36]. Dicer-independent pathways, such as those involving short hairpin RNAs (shRNAs), also exist [37]. MiRNAs play a crucial role in gene regulation, controlling mRNA expression and orchestrating transcription and translation processes [38]. Two well-defined pathways, canonical and non-canonical, contribute to miRNA synthesis. In the non-canonical mechanism, the miRISC complex, guided by miRNA strands, binds to target mRNA 3′UTR regions, initiating mRNA deadenylation, translation suppression, and degradation [39]. Approximately 60% of miRNA-mRNA interactions follow non-canonical pathways, allowing for diverse biological processes and multiple targets.

Circulating miRNAs, termed exogenous miRNAs, serve as vital signaling molecules in extracellular communication. Initially located within cells, they can be released into bodily fluids through various mechanisms, contributing to diverse health conditions [40]. Scientific progress has unveiled the origins, functions, and clinical applications of miRNAs. Specific circulating miRNAs participate in these mechanisms, as identified by prior research [41,42].

## 3 Prospective role of miRNAs as indicators and targets for therapeutic interventions in meningiomas

Kliese et al. investigated the role of miR-145–5p in meningiomas. A study found a significant decrease in miR-145–5p expression in atypical and anaplastic meningiomas compared to their benign counterparts [43]. To understand the implications, the researchers overexpressed miR-145–5p in IOMM-Lee meningioma cells, which led to several important findings. Notably, there was a marked decrease in cell proliferation, increased sensitivity to apoptosis (programmed cell death), and a decrease in anchorage-independent growth. Even more promising, overexpression of miR-145–5p resulted in decreased orthotopic tumor growth when these cells were implanted into nude mice compared to control cells. Additionally, the study found that meningioma cells with elevated levels of miR-145–5p exhibited impaired migratory and invasive potential both in vitro and in vivo. This suggests a potentially important role for miR-145–5p in reducing the spread and invasiveness of these tumors. Interestingly, PCR studies showed that overexpression of miR-145–5p suppresses the expression of collagen type V alpha (COL5A1). Importantly, the researchers also found that COL5A1 expression is significantly increased in atypical and anaplastic meningiomas. This finding provides a potential link between miR-145–5p and COL5A1 regulation in meningiomas, shedding light on a potential mechanism of tumor suppression. Taken together, this study offers compelling evidence that miR-145–5p plays a key role in limiting the migratory and proliferative capacity of meningiomas, especially aggressive variants. The study opens the door for further exploration of miR-145–5p as a therapeutic target to counter the aggressive characteristics associated with atypical and anaplastic meningiomas [43].

**Fig. 2:**
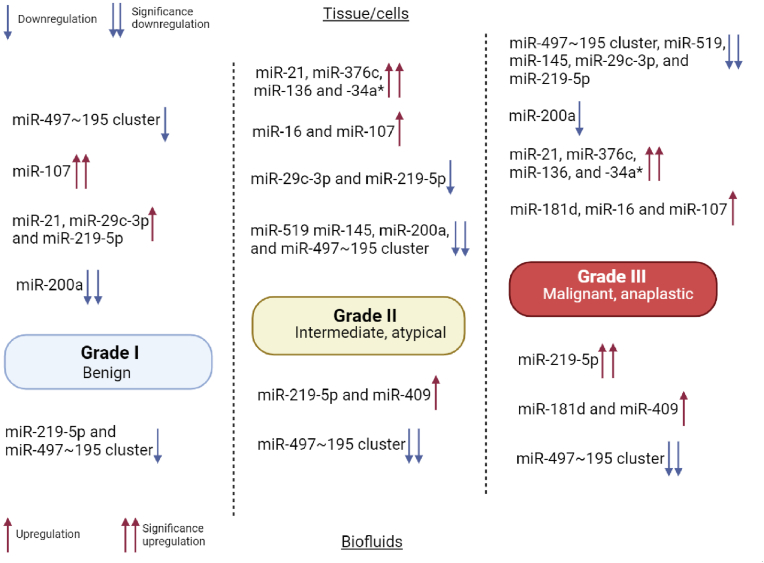
Depicts the correlation between the expression of microRNAs (miRNAs) and the histopathologic grade of meningioma. Meningiomas are categorized into Grades I–III based on the WHO grading system, and the profiles of miRNAs in tissues/cells and biofluids exhibit associations with various meningioma grades.

Dalan et al. used an integrative approach combining miRNA and mRNA transcriptome analysis to unravel the molecular mechanisms contributing to the initiation and progression of meningiomas [44]. The research methodology involved the analysis of fresh frozen samples of human meningioma and meningeal cell lines. MicroRNA and whole transcriptome microarrays were used to study these samples. Subsequent data were subjected to rigorous filtering and analysis, resulting in the identification of dysregulated genes and microRNAs. Notably, this approach was followed by a validation phase on a larger cohort of fifty-eight patient samples. The study identified a significant number of genes (3753) and microRNAs (891) that are dysregulated in meningiomas, shedding light on the complex molecular landscape of these tumors [44]. When these results were combined and analyzed using bioinformatics tools, several major differential pathways emerged, including those related to inflammation, cancer, cell growth, and cell survival. In addition, the study revealed significantly low expression of the tumor suppressor PTX3 in meningioma samples. Importantly, PTX3 showed a negative correlation with miR-29c-3p in the samples studied. The study took another step forward in exploring the functional relationship between miR-29c-3p and PTX3. Inhibition of miR-29c-3p resulted in upregulation of PTX3, which in turn caused apoptosis of meningioma cells and decreased cell viability. This functional validation strengthens the association between miR-29c and PTX3 in the context of meningiomas. Moreover, the study identified several genes, including CABIN1, TMOD1, RPL22, SPARCL1, and RELA, that were correlated with clinicopathological features in patient samples. These findings may serve as valuable clinicopathological markers to aid in patient management and prognosis (Fig. 2).

Saidam et al. focused on a specific microRNA, miR-200a-3p, and its role in the development and progression of meningioma [45]. The researchers began their study by profiling the microRNA landscape of typical human meningiomas. Notably, they found that miR-200a-3p expression was reduced in these tumors. To understand the impact of this suppression, the study performed experiments in both cell culture and an in vivo tumor model. The results were amazing. Increasing miR-200a-3p levels had a significant inhibitory effect on the growth of meningioma cells. This discovery alone highlights the potential of miR-200a-3p as a significant player in suppressing meningioma development. The research then delved into the molecular mechanisms at play. MiR-200a-3p was found to affect the expression of key transcription factors ZEB1 and SIP1, which in turn affect the expression of E-cadherin, a critical adhesion protein associated with cell differentiation. This indicates that miR-200a plays a multifaceted role in regulating cell growth and differentiation in meningiomas. A key finding in this study was the direct targeting of beta-catenin mRNA by miR-200a-3p. Beta-catenin is a protein associated with the Wnt/beta-catenin signaling pathway that is often involved in various cancers, including meningiomas. By inhibiting beta-catenin translation, miR-200a-3p effectively blocks the Wnt/beta-catenin signaling pathway, which is an important step in cancer progression. The researchers then confirmed their findings in human meningioma samples, finding a direct correlation between decreased miR-200a-3p levels and increased beta-catenin levels. These clinical data confirmed the importance of miR-200a-3p as a multifunctional tumor suppressor microRNA in meningiomas. This study reveals a complex and previously unrecognized signaling cascade involved in the development of meningioma. The multifaceted role of miR-200a-3p in regulating the E-cadherin and Wnt/beta-catenin signaling pathways provides greater insight into the molecular mechanisms driving these tumors. Moreover, the findings open new avenues for potential therapeutic interventions, highlighting the potential of miR-200a-3p-based therapies in the fight against meningiomas [45].

Abdelrahman et al. introduced a new concept of using circulating miRNAs, particularly miR-497–5p and miR-219a-5p, as potential biomarkers to improve the diagnosis and classification of meningioma [46]. The researchers set out to examine serum and exosomal levels of miR-497–5p in a large sample of meningioma cases spanning WHO grades I to III, as well as in a group of healthy individuals. In addition, they examined the serum levels of miR-219a-5p in patients with grade I to III meningioma. MiR-497–5p demonstrated the ability to distinguish meningioma cases from control samples, offering a potential diagnostic tool for these tumors. More importantly, miR-497–5p was found to be a suitable indicator for the classification of meningiomas, which is a critical aspect of patient management. The combination of miR-497–5p and miR-219a-5p improved diagnostic performance, superior to using either miRNA individually for meningioma classification. This innovative approach to combining biomarkers has the potential to improve the accuracy of meningiomas classification [46]. What sets this study apart from others is the examination of the correlation between circulating serum microRNAs and methylation status in meningioma, an aspect that has not been previously studied. This provides greater insight into the molecular basis of these tumors.

Another study examined the expression levels of miR-451a and miR-885–5p in 29 meningioma patients [47]. The results revealed a significant increase in the expression of miR-451a (p = 0.003), indicating its potential significance as a biomarker. However, no significant changes in miR-885–5p expression levels (p = 0.139) were observed in meningioma patients compared to controls. Notably, the expression levels of both miRNAs did not differ significantly according to the histopathological grade of meningioma. The observed upregulation of miR-451a in meningioma patients suggests its potential utility as a diagnostic marker. Its specificity in distinguishing meningioma cases from controls underscores its promise for clinical use. Additionally, the study hints at the potential prognostic value of miR-451a, highlighting its role in predicting the progression of meningioma. The identification of miR-451a as a potential biomarker opens the door to its exploration as a therapeutic target for the treatment of meningioma [47]. Further studies are needed to elucidate the underlying mechanisms by which miR-451a contributes to meningioma pathogenesis, paving the way for targeted interventions.

Carneiro et al. are exploring the potential role of miRNAs as biomarkers in the context of meningiomas, which are common intracranial tumors derived from arachnoid meningothelial cells [48]. Meningiomas constitute a significant percentage of intracranial tumors, making them a subject of significant clinical interest. The study focused on three specific miRNAs: miR-181 d, miR-181c and miR-130a, assessing their tissue and plasma expression in patients with different grades of meningioma [48]. The selection of these miRNAs is based on previous large-scale microarray analysis, reflecting the continuity and progress of research. The study included patients with grades I, II, and III meningiomas to examine potential differences in miRNA expression associated with tumor grade. MiR-181 d is markedly overexpressed in both tumor tissue and plasma in the study groups, and its expression level is positively correlated with the degree of tumor progression. In contrast, miR-181c and miR-130a did not show significant differences in expression levels between groups in either tumor tissue or plasma. The conclusion drawn from this study is that miR-181 d has the potential to act as a biomarker for meningiomas. Its overexpression, especially in correlation with higher tumor grades, suggests its significance as an indicator of disease severity and progression.

MiR-224–3p is a known player in various malignancies and is associated with adverse clinical outcomes. One study aimed to examine the expression of miR-224–3p in meningiomas, its role in disease progression, and its potential as a prognostic marker [49]. The study focused on exploring the correlation between miR-224–3p expression and clinicopathological features, as well as its prognostic value in meningioma patients [49]. MiR-224–3p is significantly overexpressed in meningioma tissues compared to normal brain tissues. Moreover, its expression levels are positively correlated with the degree of tumor pathology. Kaplan-Meier analysis revealed a key association between miR-224–3p expression and patient outcomes. Meningioma patients with lower miR-224–3p expression have increased overall survival and disease-free survival, highlighting the potential of miR-224–3p as a prognostic marker. The study also delves into the biological effects of miR-224–3p on meningioma cells. Downregulation of miR-224–3p was found to suppress cell growth and enhance apoptosis, partly through interaction with the ERG2 gene, which leads to activation of the ERG2-BAK-induced apoptosis pathway. The results suggest that miR-224–3p may serve as a promising prognostic marker for meningioma patients, helping to predict overall survival and disease-free survival [49].

Zhang et al. examined the role of ferroptosis-related genes and miRNAs in meningiomas, shedding light on potential therapeutic strategies for this condition. The study uses a range of analytical techniques to uncover the molecular mechanisms at work in meningiomas and how they relate to ferroptosis, a form of cell death characterized by iron-dependent lipid peroxidation. MiR-127–5p was found to be expressed at low levels, whereas JAM3 was expressed at high levels in meningioma cells. This observation suggests a potential regulatory relationship between these molecules. Through experimental activation of miR-127–5p in meningioma cells, the study demonstrates its effect on cell cycle regulation and inhibition of cellular activity. Upregulation of miR-127–5p is associated with increased levels of lactate dehydrogenase (LDH), malondialdehyde (MDA), reactive oxygen species (ROS), and Fe2+ content, suggesting a potential role in promoting ferroptosis. The study shows that activation of miR-127–5p leads to decreased expression of the GPX4 protein, a key player in protecting cells from ferroptosis. Activation of JAM3 was found to reverse the effects of miR-127–5p activation, highlighting its role in these processes. The results of this study provide valuable insight into the mechanisms of meningioma formation and ferroptosis, suggesting that miR-127–5p plays a critical regulatory role through interaction with JAM3 [50].

Another study identified several potential miRNAs with significant changes in expression compared to healthy controls. These miRNAs include miR-106a-5p, miR-219–5p, miR-375–3p, miR-409–3p, miR-197–3p, and miR-224–3p [51]. To ensure the reliability and consistency of their findings, the researchers are testing the expression of these candidate microRNAs in two independent cohorts of meningioma patients and controls using quantitative reverse transcription-polymerase chain reaction (qRT-PCR). The results indicate that a panel of six serum miRNAs could potentially serve as an adjuvant diagnostic tool for meningioma patients. The area under the ROC curve (AUC) of 0.778 suggests that this panel can effectively differentiate between patients and healthy controls. The study also identifies potential clinical correlations with specific microRNAs. For example, miR-224–3p expression levels are associated with gender, and miR-219–5p expression is positively correlated with clinical stages of meningioma. Moreover, high miR-409–3p expression and low miR-224–3p expression are associated with higher relapse rates, highlighting their prognostic value [51].

Zhi et al. found that decreased levels of miR-29c-3p and miR-219–5p were associated with advanced clinical stages of meningioma [52]. This suggests that these miRNAs may serve as indicators of disease progression. Kaplan-Meier analysis shows that high expression of miR-190a and low expression of miR-29c-3p and miR-219–5p are significantly correlated with higher relapse rates in meningioma patients. This makes these miRNAs potential prognostic markers. Cox proportional hazards regression analysis highlights that miR-190a expression level is an independent prognostic predictor that is not influenced by other clinicopathological factors. This finding highlights the clinical relevance and prognostic value of miR-190a in predicting postoperative outcomes. In conclusion, this study's comprehensive approach to miRNA profiling in meningiomas highlights several miRNAs that exhibit significantly different expression profiles compared to normal adjacent tissue. These miRNAs, especially miR-190a, miR-29c-3p, and miR-219–5p, show potential as effective diagnostic and prognostic markers for meningiomas.

Another study provides valuable information on predicting meningioma recurrence using molecular genetic markers, focusing on microRNA (miRNA) expression. The growth rate of meningioma can vary significantly even within benign subgroups [53]. Accurate prediction of the likelihood of relapse is important for planning appropriate therapy. In a large cohort of 172 patients, miR-15a-5p, miR-146a-5p, and miR-331–3p were the most significant predictors of time to relapse [53]. Subsequent validation steps highlighted the critical importance of miR-146a-5p and miR-331–3p. Clinical factors such as extent of tumor resection and WHO grade were also considered in prognostic models. The final prognostic model developed on the expansion cohort showed that lower miR-331–3p expression and partial tumor resection were significant prognostic factors. In cases of total tumor resection, these miRNAs remained important in prognostic models, even after adjusting for clinical factors. These prognostic models can more accurately predict the timing of meningioma recurrence.

**Fig. 3:**
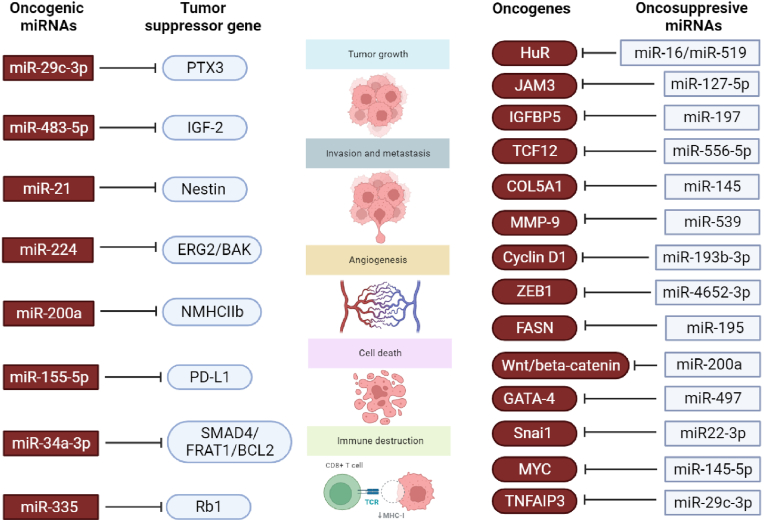
Presents a compilation of microRNAs (miRNAs) acting as either oncogenes or tumor suppressors, influencing gene targets in meningioma. This provides a basis for formulating hypotheses regarding the diverse roles played by miRNAs in modulating the characteristic features of meningioma symptoms. The findings from the research suggest a scenario wherein the transcriptome of cancer cells, primarily influenced by dysregulated tumor suppressor genes and oncogenes, is predominantly under the regulatory control of miRNAs. An example is the miR-200a, a member of the miR-200 family, which exhibits a dual role as either a tumor suppressor or an oncogene in meningioma.

Song et al. explore the pathogenesis of malignant meningioma, focusing on the role of fatty acid synthase (FASN) and miR-195–5p [54]. The study used RNA sequencing and miRNA microarray analysis to identify differentially expressed mRNAs and miRNAs in benign and malignant meningiomas. Among the identified genes, FASN stood out, the activity of which is significantly increased in malignant meningioma. A study showed that miR-195–5p directly targets FASN, leading to its downregulation [54]. Notably, upregulation of miR-195–5p inhibits proliferation, migration, and invasion of meningioma cells. This suggests that miR-195–5p plays a tumor suppressive role in the development and progression of malignant meningioma. Bioinformatics analysis was performed to predict the presence of competing endogenous RNAs (ceRNAs) that may regulate FASN expression by penetrating miR-195–5p in meningioma. The study identified several potential ceRNAs, including NUP210, SPIRE2, SLC7A1, and DMTN. This study sheds light on the pathogenesis of malignant meningiomas, highlighting the role of FASN and miR-195–5p [54]. The results highlight the tumor suppressive function of miR-195–5p by targeting FASN, which influences cell proliferation, migration, and invasion. Additionally, the study introduces the concept of ceRNAs, including NUP210, SPIRE2, SLC7A1, and DMTN, which may interact with miR-195–5p to modulate FASN expression. These data provide the basis for potential therapeutic strategies targeting FASN and miR-195–5p in the treatment of malignant meningioma, offering hope for improved clinical outcomes (Fig. 3).

Shi et al. investigated the role of miR-335–5p in meningiomas, one of the most common benign brain tumors [55]. Prevalence of miR-335–5p in meningiomas. The study shows that miR-335–5p is often overexpressed in human meningiomas. This finding suggests that miR-335–5p may play a significant role in the pathogenesis of these tumors. It has been experimentally shown that increased levels of miR-335–5p lead to increased cell growth. Moreover, overexpression of miR-335–5p inhibits cell cycle arrest at G0/G1 phase. Conversely, reducing miR-335–5p levels has the opposite effect, slowing tumor growth and progression. Previous studies have established a link between miR-335–5p and the human retinoblastoma 1 (Rb1) signaling pathway. In this context, miR-335–5p appears to influence meningioma cell proliferation by directly affecting the Rb1 pathway. A study suggests that miR-335–5p may serve as a potential therapeutic agent targeting the proliferation of meningioma cells [55]. Understanding its role in promoting cell growth and inhibiting cell cycle arrest, miR-335–5p emerges as a promising candidate for developing new treatment strategies for meningiomas. Study reveals previously unrecognized molecular interaction between miR-335–5p and Rb1. This interaction is critical for promoting the proliferation of meningioma cells. Thus, the effect of miR-335–5p on the Rb1 pathway represents an important discovery in understanding the molecular mechanisms of meningioma development. The study highlights the importance of miR-335–5p in promoting meningioma cell proliferation and the potential therapeutic implications of targeting this miRNA.

In a separate investigation, researchers explored the potential of selected microRNAs (miRNAs) as indicators of meningioma progression and recurrence [56]. Despite their initial benign nature, meningiomas can exhibit recurrence in approximately 20% of cases following surgical resection, emphasizing the need for markers to identify tumor progression and recurrence for improved patient management. The study focused on analyzing specific miRNAs, namely miRNAs 21–3p, 34a-3p, 200a-3p, and 409–3p, in both solid tumor samples and blood samples obtained from meningioma patients [56]. This innovative approach, known as liquid biopsy, offers a non-invasive means of monitoring disease progression. Results from the study revealed that miRNA 200a-3p exhibited significantly lower expression in recurrent meningiomas compared to newly diagnosed cases. This finding suggests the potential of miRNA 200a-3p as a valuable biomarker for predicting meningioma recurrence. Furthermore, the study delved into the relationship between microRNA expression and chromosomal aberrations. Notably, miRNA 409–3p demonstrated a significant correlation with chromosome 14 aberrations. Understanding these associations may contribute to a more comprehensive understanding of the biology of meningioma. The study implies that microRNA 200a-3p, particularly in blood samples, could serve as a liquid biopsy marker for identifying patients with meningioma. This non-invasive method holds great promise for monitoring and following up with patients, offering a less burdensome alternative to traditional tissue biopsy. The potential application of liquid biopsy in meningioma management highlights its significance as a valuable tool in clinical settings, providing clinicians with insights into disease progression and aiding in the development of personalized treatment approaches. The identification of specific miRNAs and their correlations with chromosomal aberrations adds a layer of complexity to our understanding of meningioma biology, opening avenues for further research and potential therapeutic interventions.

Senol et al. studied the role of miR-200a-3p in the pathogenesis of meningiomas [57]. The study identifies miR-200a-3p as a key regulator and highlights its potential implications for therapy. The study uses a comparative protein profiling approach using Gel-nanoLC-MS/MS, which identifies approximately 130 dysregulated proteins in meningioma cells overexpressing miR-200a-3p. This comprehensive analysis provides insight into the molecular changes associated with miR-200a-3p overexpression. The study uses bioinformatics analysis to identify potential target genes of miR-200a-3p. Non-muscle heavy chain IIb (NMHCIIb) is emerging as a promising target, and this protein plays a key role in cell division and migration [57]. The study shows that miR-200a-3p directly targets NMHCIIb, providing a molecular link between the miRNA and a key protein involved in cellular processes critical to tumor progression. NMHCIIb is known for its role in regulating cell division and migration. The study examines how miR-200a-3p influences these processes by targeting NMHCIIb. Overexpression of NMHCIIb was shown to partially reverse the inhibition of cell migration and growth induced by miR-200a-3p. Silencing of NMHCIIb expression by siRNA results in a similar migration phenotype and inhibits tumor growth in meningioma cells both in vitro and in mouse models. This suggests that NMHCIIb may be a promising therapeutic target for the treatment of meningiomas. These results shed light on the potential of NMHCIIb to inhibit the growth and migration of meningioma cells [57].

Another study found high and grade-dependent expression of miR-483–5p in meningioma tumor samples, which correlated with increased mRNA and protein levels of its host gene IGF-2 [58]. Inhibition of miR-483–5p demonstrated decreased growth of cultured meningioma cells, whereas miR-483 mimicked increased cell proliferation. *Anti*-IGF-2 neutralizing antibodies are also effective in reducing the proliferation of meningioma cells. Importantly, blockade of the IGF-2 receptor (IGF1R) tyrosine kinase inhibitor with a small molecule inhibitor resulted in a rapid loss of viability of cultured meningioma cells. The results showed that IGF-2 autocrine feedback is critical for the survival and growth of meningioma tumor cells [58]. This study highlights the critical dependence of meningioma cell growth on the autocrine miR-483/IGF-2 stimulation pathway. Notably, the IGF-2 pathway is emerging as a promising and feasible therapeutic target for the treatment of meningioma. Inhibition of miR-483–5p, *anti*-IGF-2 neutralizing antibodies, and small molecule tyrosine kinase inhibitors targeting IGF1R have demonstrated efficacy in limiting meningioma cell proliferation in vitro [58]. The observed inhibitory effects of GSK1838705 A and ceritinib on IGF1R, combined with pharmacokinetic data, suggest potential in vivo applications as novel treatments for meningioma.

Hergalant et al. found that tumor tissues showed decreased levels of miR-16–5p and hsa-miR-519a-5p compared to arachnoid cells from healthy patients [59]. Individual overexpression of these miRs in Ben-Men-1 and IOMM-Lee resulted in decreased cell growth. Transcriptomic analysis of IOMM-Lee cells revealed downregulation of genes, including ELAVL1/HuR, associated with mitotic cell cycle regulation, the prereplicative complex, and brain development. A specific transcriptomic signature of miR-16–5p/hsa-miR-519a-5p dysregulated genes highly enriched in HuR targets was identified. These data suggest a putative tumor suppressor effect of miR-16–5p and hsa-miR-519a-5p in meningiomas, mediated, at least in part, by inhibition of HuR, a key regulator of RNA stability and translation. The identified transcriptomic signature strengthens the association between these miRNAs and HuR targets, hinting at their critical role in modulating biological processes associated with meningioma pathogenesis. The robustness of the study was further confirmed by screening publicly available transcriptome and miRNA datasets from human meningiomas, highlighting the potential clinical relevance of miR-16–5p and hsa-miR-519a-5p as therapeutic targets for meningioma treatment [59]. Fig. 3 shows meningioma-related miRNAs (Fig. 3).

## 4 Conclusion

The investigation into miRNAs within meningiomas has yielded invaluable insights, highlighting their potential as biomarkers and targets for therapeutic interventions. MiRNAs emerge as promising diagnostic biomarkers for meningiomas, showcasing distinct expression patterns in meningioma tissues compared to normal brain tissue. This divergence opens up possibilities for early and precise tumor detection, potentially leading to enhanced patient outcomes through timely intervention. Specific miRNAs have been identified with ties to the prognosis of meningioma patients, serving as predictive indicators for tumor recurrence and progression. The recognition of these miRNAs provides clinicians with the tools to customize treatment plans and surveillance strategies for individual patients. MiRNAs introduce a novel strategy for developing targeted therapies in meningiomas. Modulating specific miRNAs holds the potential to inhibit tumor growth, induce apoptosis, or augment the sensitivity of meningioma cells to conventional treatments such as surgery or radiation therapy. This paves the way for more effective and less invasive therapeutic options. The intricate landscape of miRNA expression in meningiomas underscores the heterogeneity of these tumors, where various subtypes and grades may exhibit unique miRNA signatures. This knowledge informs personalized treatment strategies and underscores the importance of understanding this diversity for improved patient outcomes. This article emphasizes the imperative need for ongoing research into miRNAs in meningiomas. Future studies should focus on validating the diagnostic and prognostic value of specific miRNAs in larger patient cohorts. Moreover, exploring targeted therapies based on miRNA modulation is crucial, with the aim of translating these findings into clinical applications. In summary, miRNAs in meningiomas hold substantial promise as both diagnostic biomarkers and therapeutic targets, offering potential advancements in early detection, prognosis assessment, and personalized treatment development in the field of neuro-oncology. Continued research and clinical validation are essential steps toward realizing the full potential of miRNAs in managing meningiomas.

## Funding

This work was supported by the Bashkir State Medical University Strategic Academic Leadership Program (PRIORITY-2030).

## CRediT authorship contribution statement

**Ozal Beylerli:** Supervision, Conceptualization. **Tatiana Ilyasova:** Writing – review & editing. **Huaizhang Shi:** Writing – review & editing, Project administration. **Albert Sufianov:** Writing – review & editing, Conceptualization.

## Declaration of competing interest

Ozal Beylerli is an editorial board member for Non-coding RNA Research and was not involved in the editorial review or the decision to publish this article. All authors declare that there are no competing interests.
